# Nonmetabolic functions of metabolic enzymes in cancer development

**DOI:** 10.1186/s40880-018-0336-6

**Published:** 2018-10-26

**Authors:** Sean Lu, Yugang Wang

**Affiliations:** Brain Tumor Center and Department of Neuro-Oncology, The University of Texas MD Anderson Cancer Center, Houston, TX 77030 USA

**Keywords:** Metabolic enzymes, Function, Protein kinase, Metabolite products, Phosphorylation, Acetylation, Succinylation, Mitochondria

## Abstract

Metabolism is a fundamental biological process composed of a series of reactions catalyzed by metabolic enzymes. Emerging evidence demonstrates that the aberrant signaling in cancer cells induces nonmetabolic functions of metabolic enzymes in many instrumental cellular activities, which involve metabolic enzyme-mediated protein post-translational modifications, such as phosphorylation, acetylation, and succinylation. In the most well-researched literatures, metabolic enzymes phosphorylate proteins rather than their metabolites as substrates. Some metabolic enzymes have altered subcellular localization, which allows their metabolic products to directly participate in nonmetabolic activities. This review discusses how these findings have deepened our understanding on enzymes originally classified as metabolic enzymes, by highlighting the nonmetabolic functions of several metabolic enzymes responsible for the development of cancer, and evaluates the potential for targeting these functions in cancer treatment.

## Introduction

Metabolism, regulated by metabolic enzymes, supports cell growth and proliferation by providing energy for cellular activities and synthesizing the molecular building blocks for production of amino acids, nucleotides, and lipids. Metabolic enzymes are responsible for specific chemical reactions on metabolites, which takes place under strict regulation at specific subcellular locations in the metabolic cascades. Metabolic enzymes, such as carboxylases, dehydrogenases, lipoxygenases, oxidoreductases, kinases, lyses, and transferases carry out a wide range of catalytic activities and are responsible for a variety of cellular functions necessary for cellular homeostasis and survival.

Recent literatures have determined that some metabolic enzymes possess nonmetabolic activities that are critical in the development of cancer. These nonmetabolic activities can be divided into two categories. First, metabolic enzymes that use non-metabolites as substrates to catalyze reactions that are distinct from the metabolic reactions in which they were originally characterized to work on. For instance, several metabolic enzymes use proteins as substrates and function as protein kinases to phosphorylate these protein substrates, thereby regulating diverse functions [1]. Second, metabolic enzymes that translocate from their original subcellular compartments to different organelles, where their metabolite products are directly used for protein modifications or acting as instrumental regulators for other proteins. For instance, mitochondrial α-ketoglutarate dehydrogenase (α-KGDH) that translocates to the nucleus and produces succinyl-coenzyme A (CoA), which is used by the histone acetyltransferase, lysine acetyltransferase 2A (KAT2A), to succinylate histone H3 [2, 3]. In addition, mitochondrial fumarase, when translocated to the nucleus, produces fumarate that inhibits lysine demethylase 2B (KDM2B) histone demethylase activity and enhances the methylation of histone H3 and the repair of damaged DNA [4]. This review summarizes the recent findings regarding these nonmetabolic functions of metabolic enzymes and highlights the implication of these functions in cancer development.

## Metabolic enzymes function as protein kinases

Protein kinases are critical regulators of intracellular signal transduction pathways that mediate various cellular processes in both unicellular and multicellular organisms. They can directly transfer the γ-phosphate from adenosine triphosphate (ATP) to specific tyrosine (Tyr), serine (Ser), threonine (Thr), and histidine (His) residues on substrate proteins, thereby altering the functions of these substrates. More than 500 protein kinases have been identified in humans, constituting of about 1.7% of all human genes [5]. Recent studies have demonstrated that several metabolic enzymes, such as pyruvate kinase M2 (PKM2), phosphoglycerate kinase 1 (PGK1), ketohexokinase-A (KHK-A), hexokinases (HK), nucleoside diphosphate kinase (NDPK or NDK), and 6-phosphofructo-2-kinase/fructose-2,6-biphosphatase 4 (PFKFB4), have unexpected protein kinase activities and play significant roles in nonmetabolic cellular functions. These new studies expand the family of protein kinases and provide new insights into the integrated regulation of cell metabolism and other cellular processes.

### PKM2

Pyruvate kinase (PK) catalyzes the final rate-limiting step of glycolysis and converts phosphoenolpyruvate (PEP) to pyruvate by transferring a phosphate group from PEP to adenosine diphosphate (ADP), producing ATP. It has four isoforms: PKL, PKR, PKM1, and PKM2. PKM2 is highly expressed in cancer cells [6]. Besides, although PKM1 has a higher glycolytic activity than PKM2, only the protein kinase activity of PKM2 has been described till present. PKM2 is involved in the regulation of gene expression, mitosis, apoptosis, and other critical cellular activities that promote aerobic glycolysis and tumor growth [7–9].

PKM2’s protein kinase activity was initially identified from the phosphorylation of histone H3 at Thr11 and that of signal transducer and activator of transcription 3 (STAT3) at Tyr705. In the nucleus, PKM2-mediated histone H3 phosphorylation promotes β-catenin- and c-Myc-mediated gene expression, which enhances aerobic glycolysis and promotes the proliferation of tumor cells [10–14]. During mitosis, PKM2 binds to the spindle checkpoint protein Bub3 and phosphorylate it at Tyr207 to enable the interaction of the Bub3–Bub1 complex with kinetochores, which is essential for the mitotic/spindle-assembly checkpoint, accurate chromosome segregation, and tumorigenesis [15]. PKM2 also phosphorylates myosin light chain 2 (MLC2) at Tyr118, primes the binding of Rho-associated protein kinase 2 (ROCK2) to MLC2 and the phosphorylation of ROCK2–MLC2 complex at Ser15, to allow the interaction between myosin II with actin, which is required for the contractile function of the actomyosin complex at the cleavage furrow, completion of the cytokinesis process, and proliferation of tumor cells [16]. In addition, it has been found that in hepatocellular carcinoma (HCC), PKM2 phosphorylates the sterol regulatory element-binding proteins (SREBPs) at Thr59, activates lipid biosynthesis, and promotes the proliferation of the cancer cells [17]. Apart from its functions in regulating gene expression and cell cycle progression, PKM2 has been demonstrated to phosphorylate serine/threonine kinase 1 (AKT1) at Ser202/203 to release it from the regulatory-associated protein of mTOR (raptor), which subsequently promotes hormonal and nutrient signaling-independent activation of the mammalian target of rapamycin complex 1 (mTORC1), to provide survival and proliferation advantages over normal cell upon stimulus by the epidermal growth factor receptor (EGFR)-dependent activation of nuclear factor kappa enhancer binding protein (NF-κB) [6, 18].

In response to oxidative stress, PKM2 promotes cell survival by translocating into the mitochondria, phosphorylating the apoptosis regulator Bcl2 at Thr69 to stabilize Bcl2, which blocks its association with Cul3-based E3 ligase to prevent the degradation of Bcl2 and promotes the resistance of tumor cells against apoptosis [19]. Further, as PKM2 can also phosphorylate the synaptosome-associated protein 23 (SNAP23) at Ser95, resulting in the formation of the soluble *N*-ethylmaleimide sensitive factor attachment protein receptors (SNARE) complex, this demonstrates that PKM2 has contribution in the remodeling of the tumor microenvironments by promoting tumor cell exosome secretions, which are necessary for the docking of tumor cells to plasma membranes [20]. A recent report has shown that in pancreatic ductal adenocarcinoma, PKM2 could phosphorylate the serine/threonine protein kinase PAK2 at Ser20, Ser141, and Ser 192/197 to recruit heat shock protein 90 for increasing the stability of the PAK2 protein, thereby promoting invasion and metastasis of the pancreatic cancerous cells [21]. Further, PKM2 was observed to promote genomic instability, an important hallmark of cancer development and progression due to increased interruption of DNA repair, in breast cancer cells breast cancer since it was demonstrated that nuclear PKM2 could interact with and directly phosphorylate histone H2AX at Ser139 under DNA damaged conditions to induce chromosomal aberrations and promote cancer cell proliferation [22]. Furthermore, phosphoproteomic studies in yeast and mammalian cells have revealed that hundreds of additional proteins, including many protein kinases such as extracellular signal-regulated kinase (ERK), AKT1 substrate 1 (AKT1S1), BLK, can be phosphorylated by PKM2 [18, 23, 24], suggesting that PKM2’s protein kinase activities have instrumental roles in a wide array of cellular functions.

### PGK1

PGK1 is an enzyme responsible for the first ATP-generating step in the glycolysis pathway and is highly expressed in many types of cancer [25]. It catalyzes the reversible conversion of 1,3-diphosphoglycerate and ADP to 3-phosphoglycerate and ATP, respectively [25]. And similar to PKM2, PGK1 also has protein kinase activity to phosphorylate protein substrates. We found that mitochondria-translocated PGK1 uses ATP as a phosphate donor to directly phosphorylate and activate pyruvate dehydrogenase kinase isozyme 1 (PDHK1) at Thr338. The subsequent PDHK1-mediated phosphorylation of pyruvate dehydrogenase E1α at Ser293 inhibits the pyruvate dehydrogenase complex (PDC) and the conversion of pyruvate and CoA to acetyl-CoA in the mitochondria, which suppresses the mitochondrial pyruvate oxidation and increases lactate production from pyruvate in the cytosol. Thus, mitochondrial pyruvate metabolism suppressed by PGK1 couples with the upregulation of glycolytic gene expression mediated by nuclear PKM2 to promote the Warburg effect and tumorigenesis [26]. In addition, the protein kinase activity of PGK1 is involved in the initiation of autophagy, important for homogenizing cell homeostasis. When tumors start outgrowing existing vasculature, this result in glutamine deprivation and hypoxia within the tumor cells and ARD1 acetyl-transferase acetylates PGK1 at Lys388, which in turn phosphorylates Beclin1 at Ser30, leading to a conformational change and activation of class III phosphatidylinositol (PI) 3-kinase (VPS34) to produce phosphatidylinositol 3-phosphate (PI(3)P), facilitating the initiation of tumor-autophagy and promotes tumor development [27]. Thus, targeting PGK1 can be an attractive therapeutic approach for cancer treatment.

### KHK-A

KHK, also known as fructokinase, is responsible for the first rate-limiting enzymatic reaction in fructose metabolism. KHK catalyzes the transfer of a phosphate group from ATP to fructose, producing fructose 1-phosphate (F1P) and ADP. Aldolase then catalyzes the F1P into dihydroxyacetone phosphate and glyceraldehyde, which are subsequently converged into the glycolysis pathway [28]. Among the alternatively spliced isoforms of the precursor ribonucleic acid (RNA) of KHK, highly active KHK-C, but not inactive KHK-A, is highly expressed in the liver, kidneys, and pancreas [29]. In HCC cells, c-Myc induces the expression of heterogeneous nuclear ribonuclear proteins H1 (HnRNPH1) and nRNPH2, which regulates the splicing of the KHK precursor mRNA and switches KHK-C to KHK-A. The expression of KHK-A, which has a much lower activity toward phosphorylation of fructose, slows the fructose catabolism rates, ATP consumption, and reactive oxygen species production in HCC cells [30]. Importantly, instead of binding to fructose, KHK-A interacts with the rate-limiting enzyme phosphoribosyl pyrophosphate synthetase 1 (PRPS1) in the de novo nucleic acid synthesis pathway and acts as a protein kinase to directly phosphorylate PRPS1 at Thr225. This phosphorylation blocks the ADP binding-mediated inhibition of PRPS1, resulting in elevated de novo nucleic acid synthesis via the constitutive activation of PRPS1 and enhancing HCC cell proliferation and liver tumor growth in mice. KHK-A expression and PRPS1 Thr225 phosphorylation levels are positively correlated with each other in human HCC specimens and are inversely correlated with survival in HCC patients, indicating that KHK-A-dependent PRPS1 phosphorylation is pivotal in HCC progression [28, 30].

### HK

Most cancers ensure sufficient energy supplies via glycolysis which are the basis for their growth and proliferation. However, the systemic inhibition of glycolysis as an anti-cancer approach would result in considerable adverse effects since normal cells also supplements themselves with energy through glycolysis. Therefore, the selective inhibition of cancer-driven glycolysis has been investigated for clinical cancer therapy and HK was proposed as a therapeutic target. HK enzymes have been found to be essential in the first step of the glucose metabolism pathway as they catalyse the phosphorylation of glucose to produce glucose 6-phosphate by using ATP as the phosphate donor. Phosphoamino acid analyses revealed that HK1 purified from rat brains can be autophosphorylated at serine, threonine, and tyrosine residues [31], and in vitro phosphorylation assays showed that HK1 can phosphorylate itself and purified histone H2A [32]. However, the different isoforms of HK need to be characterized and whether HK1 or other HK isoforms act as protein kinases in vivo remains unclear, as does the physiological role of any such phosphorylation activity in the regulation of cellular activities associated with cancer development.

### NDPK1/2

NDPK is a ubiquitous enzyme in mammals. It catalyzes the conversion of nucleoside diphosphates (NDPs) into nucleoside triphosphates (NTPs) by transferring the γ-phosphate group from the 5′-triphosphate nucleotides to the 5′-diphosphate nucleotides [33]. In this process, NDPK uses an NTP (usually ATP) and autophosphorylate itself at a highly conserved histidine residue in its active site. The phosphate group is then transferred from the phosphohistidine to an NDP molecule or to a histidine on the substrate protein [34]. Several proteins have been identified as substrates of NDPK-A and NDPK-B protein kinase activity. For instance, NDPK-A phosphorylates the histidine at the catalytic site of ATP citrate lyase (ACLY) and regulates ACLY-dependent acetyl-CoA production for fatty acid synthesis [35]. NDPK-B can form a complex with G protein βγ dimers and phosphorylate the Gβ subunit at His266. The phosphate group is then transferred to guanosine diphosphate (GDP), leading to the formation of guanosine triphosphate (GTP) and the activation of G protein [36]. NDPK-B also phosphorylates the Ca^2+^-activated K^+^ channel KCa3.1 at His358. This phosphorylation relieves the copper-dependent inhibition of KCa3.1 channel function and promotes subsequent activation of CD4^+^ T cells [37, 38]. In addition, NDPK-B phosphorylates the C-terminal tail of transient receptor potential vanilloid 5 at His711 and regulates urinary Ca^2+^ excretion by mediating active Ca^2+^ reabsorption in the distal convoluted tubule of the kidney [39]. However, like that of HK, the role of the protein histidine-kinase activity of NDPK in the development of cancer are yet to be uncovered.

### PFKFB4

In humans, four genes encode phosphofructokinase two proteins: *PFKFB1*, *PFKFB2*, *PFKFB3*, and *PFKFB4*. These proteins vary dramatically in their tissue expression, regulation, and kinase-to-phosphatase activity. Phosphofructokinase 2 promotes glycolysis by phosphorylation to generate fructose 6-phosphate, an allosteric activator of phosphofructokinase 1. In a recent study, PFKFB4 was found to have protein kinase activity that phosphorylates steroid receptor coactivator-3 (SRC3) at the Ser857 [40], which promotes the association of SRC3 with the activating transcription factor 4 (ATF4) to induce gene expression of the enzymes transketolase, adenosine monophosphate deaminase-1 (AMPD1), and xanthine dehydrogenase (XDH), involved in the purine metabolism to promote metastasis. As such, PFKFB4-activated SRC3 activation drives glucose flux towards the pentose phosphate pathway, enhances purine synthesis, and promotes tumor progression [41].

## Metabolic enzymes regulate cellular activity via their metabolite products

Metabolic enzymes can form complexes with other proteins and regulate the functions of these proteins via their metabolite products. Our research group and others have demonstrated that nucleus-localized acetyl-CoA synthetase short-chain family member 2 (ACSS2), ACLY, PDC, α-KGDH, and fumarase are in complex with chromatin modulators and regulate gene expression and DNA repair through their metabolite products.

### ACSS2

Histone acetylation requires nuclear acetyl-CoA and is critical for gene expression. ACSS2 is a non-mitochondrial source of acetyl-CoA, which localizes in the cytosol and the nucleus, and catalyzes the ligation of acetate, both from exogenous sources and recycled histone deacetylase reactions, to CoA to produce acetyl-CoA [42, 43]. In response to stresses such as hypoxia the amount of nutrients becomes limited, conditions commonly observed in tumor microenvironments, and cytosolic ACSS2 is translocated to the nucleus, where it forms a complex with transcription factor EB (TFEB), locally producing acetyl-CoA for histone H3 acetylation in the promoter regions of TFEB-regulated autophagosomal and lysosomal genes. The expression of these genes promotes glucose deprivation-induced autophagy and lysosomal biogenesis, tumor cell survival, and proliferation [43].

### ACLY

ACLY, which catalyzes the conversion of oxidation-derived mitochondrial citrate into acetyl-CoA and oxaloacetate, provides an important source of cytosolic acetyl-CoA. Nevertheless, ACLY is also found in the nucleus, and the spatial and temporal control of nuclear ACLY-dependent acetyl-CoA production plays an important role in histone acetylation and regulation of gene expression [44–46]. The identification of co-regulators that are in complex with ACLY will shed further light on the selective regulation of gene expression.

### PDC

In addition to ACLY and ACSS2, PDC, which is primarily located in the mitochondria, is another non-mitochondrial source of acetyl-CoA. Upon treatment of cells with serum and epidermal growth factor, PDC translocates into the nucleus and generates acetyl-CoA for histone acetylation [47]. As with ACLY, the complexed proteins with PDC that control gene expression remain undetermined.

### α-KGDH

Mitochondrial α-KGDH catalyzes the conversion of α-ketoglutarate into succinyl-CoA in the tricarboxylic acid cycle. In our recent studies, we have demonstrated that a small percentage of α-KGDH is located in the nucleus [2]. Nuclear α-KGDH interacts with KAT2A to form a high-order assembly and locally converts α-ketoglutarate and CoA into succinyl-CoA, which can be used by KAT2A to succinylate histone H3 [2, 3]. Nuclear α-KGDH-coupled KAT2A functions as a histone succinyltransferase to regulate gene expression, which is pivotal in promoting cancer cell proliferation and tumor growth [2, 3].

### Fumarase

Fumarase catalyzes the reversible reaction of fumarate to malate in the mitochondria and cytosol. Upon induction of DNA damage by ionizing radiation, fumarase translocates from the cytosol into the nucleus. In the nucleus, DNA-dependent protein kinase (DNA-PK)-phosphorylated fumarase binds to histone H2A.Z at irradiation-induced double-strand break regions and locally produces fumarate, which inhibits α-ketoglutarate-dependent KDM2B activity. Thus, H2A.Z-complexed fumarase increases the demethylation of histone H3K36 and accumulation of the DNA-PK complex at DNA damage foci to induce nonhomologous end joining DNA repair and enhance cell survival. In addition, chromatin-associated fumarase can regulate gene transcription. Under conditions of glucose deprivation, AMP-activated protein kinase-phosphorylated fumarase binds to the transcription factor ATF2 and translocates to ATF2-regulated gene promoter regions, where fumarase-catalyzed fumarate inhibits KDM2A, increases histone H3K36 demethylation and gene expression to inhibit cell growth. Thus, the role of fumarase in DNA damage repair and regulation of gene expression depends on its post-translational modifications and interacting proteins.

## Conclusions and perspective

Recent studies have demonstrated that metabolic enzymes have nonmetabolic activity. The characterization of this activity has expanded our understanding on the integrated spatial and temporal control of metabolic and nonmetabolic processes [48, 49]. The characterization of multiple types of protein kinase activities of PKM2, PGK1, KHK-A, HK, PFKFB4, and NDPK-A/B makes the identification of the protein kinase activity of other metabolic enzymes a reasonable quest. The ability of these metabolic enzymes to transfer their phosphate groups to substrate proteins instead of that of their substrate metabolites indicates the non-ridged substrate selectivity in the chemical reactions that are catalyzed by metabolic enzymes.

In addition to these alternative enzymatic activities, altered subcellular localization also contributes to the involvement of metabolic enzymes in nonmetabolic processes [48, 50]. Metabolic reactions takes place in specific subcellular compartments to ensure that the metabolites enter into the correct metabolic cascade. Most metabolic enzyme-mediated nonmetabolic reactions occur outside the locations of the originally identified metabolic reactions. Importantly, the nonmetabolic functions of metabolic enzymes are crucial to tumorigenesis and cancer progression. Advances in our understanding of the nonmetabolic activities of metabolic enzymes would lead to the development of new and specific therapeutics for improving cancer treatments.

## Abbreviations

α-KGDH: α-ketoglutarate dehydrogenase
ACLY: ATP-citrate lyase
ACSS2: acetyl-CoA synthetase short chain family member 2
ATF4: cyclic AMP-dependent transcription factor
CoA: coenzyme A
DNA-PK: DNA-dependent protein kinase
F1P: fructose 1-phosphate
HCC: hepatocellular carcinoma
HK: hexokinase
KAT2A: histone acetyltransferase KAT2A
KDM2B: lysine-specific demethylase 2B
KHK: ketohexokinase
MLC2: myosin light chain 2
NDP: nucleoside diphosphate
NDPK: nucleoside diphosphate kinase
NTP: nucleoside triphosphate
PDC: pyruvate dehydrogenase complex
PDHK1: pyruvate dehydrogenase kinase isozyme 1
PEP: phosphoenolpyruvate
PFKFB: 6-phosphofructo-2-kinase/fructose-2,6-biphosphatase
PGK1: phosphoglycerate kinase 1
PKM2: pyruvate kinase M2
PRPS1: phosphoribosyl pyrophosphate synthetase 1
SRC3: steroid receptor coactivator 3
TFEB: transcription factor EB
